# Diseases of the Chest

**Published:** 1835-07-01

**Authors:** 


*The Pathology and Diagnosis of Diseases of the Chest; illustrated especially by a Rational Exposition of their Physical Signs. With New Researches on the Sounds of the Heart.* By CHARLES J. B. WILLIAMS, M.D., F.R.S. &c. *Third Edition, much enlarged.*—London, 1835. 8vo. pp. 213.

THIS is a new edition of a good book, made excellent by additions and corrections; and will not only form a useful part of the library of the student, but may be consulted with great advantage by the experienced physician, not yet sufficiently versed in the use of the stethoscope. The additions are very considerable: "the sections *on the ocular and manual examination of the Chest ; on expectoration ; on encephaloid disease, melanosis, &c.; on diseases of the bronchial glands ;* and the whole of Part III. *on the auscultation of the heart*, are new; and large additions have been made to the sections on *bron-*

*chitis, peripneumony, pleurisy, pneumothorax,* and *phthisis.*" (*Pref.* p. 9.)

The following extracts from the new matter may serve to show how well Dr. Williams has employed the two years which have intervened since the last edition.

"*Ocular and Manual Examination of the Chest.*

"Much useful information as to the state of the thoracic organs may often be obtained by mere inspection and manual palpation of the chest; and although the signs resulting from this method of examination are neither so numerous nor so precise as those of auscultation, they are yet very valuable, because they are easily obtained, and because they often give a general and decisive character to other more minute indications.

"In a healthy and well acting chest, the act of inspiration is performed equally by the elevation of the ribs and the descent of the diaphragm; and the eyes watching the naked chest, or the hands feeling it, will perceive its equal and uniform expansion by these means. If any disorder impede the free descent of the diaphragm, whether it be pain of this muscle or its coverings, or of some of the viscera below, or whether it be pressure on its under surface, as from abdominal dropsy, tumours, or pregnancy, an increased task will then devolve on the ribs and their muscles, and the respiration being performed principally by the heaving of the chest, is called *thoracic.* This character of respiration is obvious to the eye: and one hand applied lightly, but in close contact, on the chest, and the other on the abdomen, equally perceive it: it becomes then the next matter of inquiry, which of the above-mentioned causes is present. Again, in the converse case, which belongs more closely to our subject, pain or increased sensibility of the parietes of the chest, or of its more superficial contents, ossification of the cartilages of the ribs, and occasionally certain changes in the lungs themselves, which will be afterwards considered, make the respiration *diaphragmatic,* or *abdominal,* the ribs remaining comparatively immobile. The diseases of the chest which may render the respiration chiefly abdominal, are pleurodyne, inflammation of the costal and upper pulmonary pleura, and occasionally induration of the lung by hepatisation or tubercles. Of those which render the respiration chiefly thoracic, besides diaphragmatic pleurisy, spasmodic asthma may be reckoned, in which the diaphragm, overcome by the superior force of the bronchial muscles spasmodically contracted, is permanently drawn into the chest, causing a remarkable hollow at the scrobiculus cordis, whilst the respiration is carried on by the intercostal muscles, and others which assist them in supplemental respiratory efforts.

"The more useful indications, however, in the external examination of the chest, are those which arise from a want of correspondence, in form or in motion, between the two sides. Any disease which interferes with the respiratory act, and affecting chiefly one side, will produce an inequality obvious to sight and feeling. For this mode of examination, the patient should be placed with his chest exposed in a good light opposite to the observer, who attentively surveys the chest, and further corrects his estimate of its form and motions, by feeling with his two hands the simultaneous motions of corresponding parts on the two sides, during increased as well as ordinary acts of respiration. To determine the comparative size of the two sides of the chest, the more accurate method of measurement from the spinous process of a vertebra to the sternum may be sometimes employed. Care must be taken that the string or tape be passed over corresponding parts; and it must be held in recollection, that in healthy persons the right side is almost always slightly larger than the left.

"Now it is obvious, that any inequality or irregularity in the form or movement of the chest will imply some disease; and, with a little further attention, the same method of examination will give some general knowledge of its seat and character. Thus, if one side appears to be immobile, with a sharp pain or stitch, but without alteration of size, it may be suspected that a pleurodyne or a recent pleurisy is the disease, and prevents the respiratory movements by the pain which it would cause in the affected part. If to the immobility of the side is joined an unnatural fulness, perceptible to the eye and to mensuration, there is perhaps effusion into the pleura, as in advanced states of pleurisy, empyema, hydrothorax, and pneumothorax. If a contraction of the side is joined to the defect of movement, adhesions or the reabsorption of a pleuritic effusion of some standing, is the probable cause. If one side does not partake in the respiratory act, and yet there is neither pain nor alteration of size, it is likely that the corresponding lungs may be hepatised, or otherwise obstructed.

"The preceding signs are connected principally with the middle and lower parts of the chest. Irregularities in the movements and shape of the upper regions are perhaps more characteristic. Thus tubercular disease rarely exists to a considerable extent, without diminishing the motion of the upper ribs of the affected side; and adhering, as the diseased lung often is, to the walls of the chest, it not unfrequently causes an angularity and want of symmetry, which are very characteristic. Opposed to this is the effect of pulmonary emphysema, which gives a full and unnaturally rounded appearance to the upper parts of the chest, whilst, both by sight and tact, it can be perceived that the chest does not rise and fall to its natural capacity." (P. 12.)

### "*Diseases of the Bronchial Glands.*

"It is very common to find the bronchial glands exhibiting traces of disease even in cases where no symptoms of it had been indicated during life. The presence of the black matter, like that in the lungs, can scarcely be called a disease, since scarcely any adult is entirely free from it. But besides this, the glands are sometimes enlarged, their unstained parts being of a reddish colour; and as they have been found in this state when the adjoining parts of the lungs have been inflamed, there is reason to believe the tumefaction to be caused by inflammation. Laennec says that he has met with abscess in them in a very few instances.

"They are frequently the seat of tuberculous deposit; and I have seen instances, both in children and in the adult, of its formation in them exclusively, no other part of the body shewing any trace of tubercles. When thus tuberculous, they sometimes attain the size of a pullet's egg, and by pressure on the trachea or bronchi, or on the blood-vessels, induce dyspnœa and obstructed circulation. Dr. Carswell considers this to be not uncommon in children; and he would ascribe to it the dyspnœa occurring in them, without signs of other disease, when the chest continues to be sonorous on percussion, and the respiration everywhere audible though weak. Tuberculous matter in the bronchial glands is liable to the softening, and to the drying and cretaceous conversion which we have noticed to be its course in the lungs. The softened matter may be evacuated through the bronchi, leaving a fistulous cavity; and M. Guersent describes this as not an uncommon case in children. Dr. Carswell notices an instance of a child, in which this softening and ulceration proved fatal, by opening a branch of the pulmonary artery.

"The calcareous matter found in the bronchial glands, does not appear to be always the dry remains of tuberculous deposits; for I have seen calculous concretions in them which are obviously different from the gypsum-like matter of old tubercles, and without the enlargement which the scrofulous tubercle generally produces.

"Encephaloid disease may attack the bronchial glands, from which it sometimes extends to the tissue of the lung. A remarkable case of this kind lately occurred at St. George's Hospital. The disease constituted a tumour in the mediastinum, which involved and partially obstructed both the blood-vessels and trachea; and nearly the whole of the left lung was solidified by an infiltration of the same matter. The mixture of black matter in the lung, and the presence of two cavities, gave it a resemblance to tuberculous disease, from which it would scarcely have been distinguished but for the presence of the reddish white organized matter in the root of the lung, and which extended into, and was gradually lost in, the solid grey matter of the lobes. The symptoms in this case were partial œdema, and lividity of the face and neck, and other signs of obstructed circulation and respiration.

"The physical signs of considerable tumours of the bronchial glands, would be dulness on percussion in the upper portions of the interscapular regions, and on the spinous processes of the upper dorsal vertebræ. Signs of excavations there would be rendered doubtful by the bronchophony and, in thin subjects, pectoriloquy, naturally existing in these spots." (P. 157.)

"*Softening of the Heart* is probably sometimes caused by inflammation, but in many cases it has been found where no signs of inflammation have existed in any form, and it is therefore rather to be regarded as a modification of nutrition, perhaps the result of a cachectic state of the fluids. In carditis, or inflammation of the muscular tissue manifesting itself during life, by increased action, and sometimes pain of the heart, dyspnœa, a very quick, contracted, and hard pulse, the heart has been found in a softened state after death, the ventricles being collapsed and flabby, and being easily lacerated when squeezed or pulled. In cases not preceded by inflammatory symptoms, a greater degree of softness has been observed in the muscular substance of the heart, which is often paler than usual, and sometimes presents a yellowish tint. Laennec considered that this softening rendered the sounds of the heart more obtuse than natural; and this, the result of his experience, we should be more ready to receive, as it accords well with the view lately given of the cause of the sounds; a soft flabby muscle being insusceptible of the degree of tension capable of producing a loud clear sound. There is, however, such a variety in the sounds of a healthy heart, that the mere obtuseness of sound would only give us a suspicion of the existence of softening in those who evince signs of peculiar weakness in the organ, sometimes with attacks of angina and palpitation, during which the sounds are often much clearer. Laennec thought that a partial softening was sometimes produced in the hours of imperfect circulation which precede a lingering death, and I have lately seen a case which was found after a death of this kind." (P. 191.)

It is but justice to add, that, independently of the insertion of fresh matter, the old has been carefully revised; the finishing touches of a zealous author are to be discovered in every part of the work.

